# Multi-Omics Approaches to Discover Biomarkers of Thyroid Eye Disease: A Systematic Review

**DOI:** 10.7150/ijbs.103977

**Published:** 2024-11-11

**Authors:** Haiyang Zhang, Yuyu Zhou, Baiguang Yu, Yuyang Deng, Yang Wang, Sijie Fang, Xuefei Song, Xianqun Fan, Huifang Zhou

**Affiliations:** 1 Department of Ophthalmology, Shanghai Ninth People's Hospital, Shanghai Jiao Tong University School of Medicine, No.639, Zhizaoju Road, Huangpu District, Shanghai, China.; 2 Shanghai Key Laboratory of Orbital Diseases and Ocular Oncology, Shanghai, China.; 3 Center for Basic Medical Research and Innovation in Visual System Diseases, Ministry of Education, Shanghai, China.

**Keywords:** Thyroid eye disease, Biomarker, Multi-omics, Bioinformation

## Abstract

Thyroid eye disease (TED) is an organ-specific autoimmune disorder that significantly impacts patients' visual function, appearance, and well-being. Despite existing clinical evaluation methods, there remains a need for objective biomarkers to facilitate clinical management and pathogenesis investigation. Rapid advances in multi-omics technologies have enabled the discovery and development of more informative biomarkers for clinical use. This systematic review synthesizes the current landscape of multi-omics approaches in TED research, highlighting the potential of genomics, transcriptomics, proteomics, metabolomics, and microbiomics to uncover novel biomarkers. Our review encompasses 69 studies involving 1,363 TED patients and 1,504 controls, revealing a wealth of biomarker candidates across various biological matrices. The identified biomarkers reflect alterations in gene expression, protein profiles, metabolic pathways, and microbial compositions, underscoring the systemic nature of TED. Notably, the integration of multi-omics data has been pivotal in enhancing our understanding of TED's molecular mechanisms and identifying diagnostic and prognostic markers with clinical potential.

## Introduction

Thyroid eye disease (TED), also known as thyroid-associated ophthalmopathy (TAO) and Graves' orbitopathy (GO), is an organ-specific autoimmune disorder characterized by the enlargement of the extraocular muscles and an increase in fatty or connective tissue volume. It is the most frequent extrathyroidal manifestation of Graves' disease (GD) and occasionally occurs in patients with autoimmune thyroiditis and other thyroid diseases, resulting from a combination of genetic and environmental factors 1. The estimated incidence rate is 4.2 per 100,000 person-years. Female patients outnumber males, and it tends to occur in individuals aged 40-50 years and 60-70 years 2. The clinical manifestations of TED include upper eyelid retraction, oedema, erythema of the periorbital tissues and conjunctivae, exophthalmos, dry eye, diplopia, and strabismus, significantly affecting the quality of life of patients 3, 4.

The pathogenesis of TED is not yet fully understood, but autoantibodies to the thyroid-stimulating hormone receptor (TSH-R) and insulin-like growth factor-1 receptor (IGF-1R) are thought to play a key role 4, 5. Current disease activity scoring relies on the European Group on Graves' Orbitopathy (EUGOGO) system for clinical activity scores (CAS), which considers the presence of pain, redness, swelling, and functional impairment of specific ocular and orbital structures 6, 7. However, the pathological processes of TED extend beyond orbital inflammation and tissue remodeling to include immune and metabolic changes in peripheral blood 8-11. These changes serve as important biomarkers for a deeper understanding of the disease's complexity. For instance, intraorbital biomarkers may describe the activation of orbital fibroblasts, changes in the fibrosis of extraocular muscles, and the proliferation of adipose tissues 12. In peripheral blood, biomarkers related to inflammation, immune cell activation, and autoimmune responses may be observed, such as elevated levels of thyroid-stimulating hormone receptor antibodies (TRAb), the presence of specific antibodies, or alterations in the proportions of immune cell subsets 13. These biomarkers are valuable not only for early diagnosis and assessment of disease activity but also for predicting disease progression and treatment response. Therefore, identifying and validating these biomarkers is crucial.

With the rapid development of high-throughput sequencing technology and the popularization of public databases, multi-omics studies using bioinformatics analysis have been conducted to identify new diagnostic or therapeutic biomarkers for various diseases, including cancers and autoimmune disorders 14, 15. According to the central dogma of molecular biology, omics fields such as genomics, epigenomics, transcriptomics, proteomics, metabolomics, and lipidomics have a promising role in investigating more biomarkers and comprehensively understanding the molecular mechanisms of TED 16. For example, several microarray gene profile studies on TED have been performed to identify differentially expressed genes (DEGs) and key pathways, revealing the underlying pathological mechanisms of TED 17-20. Using mass spectrometry profiling, differences in proteins between TED patients and control subjects can be investigated, potentially serving as biomarkers for aiding the detection and prognosis of TED in the future 21-24. Furthermore, microbiomics research is indeed crucial as it sheds light on the connection between the gut microbiota and systemic immune responses that may influence the pathogenesis of TED, broadening the understanding of TED beyond the traditional central dogma and opening up new avenues for diagnosis, treatment, and potentially even prevention 25-27.

The emergence of multi-omics technologies offers a new perspective for studying TED, providing a more holistic and integrated view of the disease's pathophysiology. This comprehensive review aims to summarize the current knowledge regarding the application of multi-omics to TED, highlighting the potential for novel biomarker discovery and improved diagnostics.

## Methods

This systematic review was conducted following the Preferred Reporting Items for Systematic Reviews and Meta-Analyses (PRISMA) 2020 Statement 28. The review protocol was registered in the International Prospective Register of Systematic Reviews (PROSPERO) platform (registration number CRD42024533793). The protocol is available online at [https://www.crd.york.ac.uk/prospero/display_record.php?ID=CRD42024533793].

### Search Strategy

Two independent researchers (HYZ and YYZ) systematically searched the PubMed, Embase, and Web of Science databases from inception to February 10, 2024. All English-language publications were retrieved without restrictions on the country of origin or article type. The search strategy is provided in Supplementary Tables 1-3.

### Selection Criteria

We included only original studies, such as cross-sectional, retrospective, and prospective studies. The inclusion criteria were as follows: (a) studies involving patients with a confirmed diagnosis of TED, (b) studies employing any method of sequencing, including epigenomics, genomics, transcriptomics, microbiomics, metabolomics, and proteomics, and (c) studies comparing TED patients with a healthy control (HC) group and/or different subgroups within the TED population, such as those in the active TED phase versus the inactive TED phase. Studies were excluded if they met any of the following conditions: (a) review articles, animal studies, conference abstracts, meeting reports, studies with unclear information, and registry data, (b) studies focusing on diseases other than TED, (c) studies lacking explicit diagnostic criteria for TED, and (d) studies without sequencing results. Two researchers (HYZ and YYZ) independently selected the studies for exclusion. Conflicts were resolved through discussion to reach a consensus or by third-party arbitration.

### Data Extraction and Visualization

Data extraction was carried out independently by two researchers and subsequently double-checked. Disputes were resolved through discussion or by consulting a third specialist. Critical characteristics from all included studies were extracted and recorded. The extracted data encompassed the author, year of publication, region, sample type and size, age and sex of each group, sequencing methods, data analysis methods, and main findings.

### Risk of Bias and Quality Assessment

The quality of the studies was assessed using the tool described by Lumbreras *et al.* according to QUADOMICS 29, which was developed to evaluate quality issues specific to omics research. Detailed information is presented in Supplementary Table 4, which considers sixteen criteria. The second and fourteenth items of QUADOMICS did not apply to the included studies, and none of the studies met item 12, which indicates that they interpreted the index test results with knowledge of the reference standard. Two independent authors (YYZ and YYD) conducted the quality assessment, and discrepancies were discussed until a consensus was reached. If consensus could not be achieved, a third specialist was consulted.

## Results

### Descriptive Characteristics

**Figure 1:**
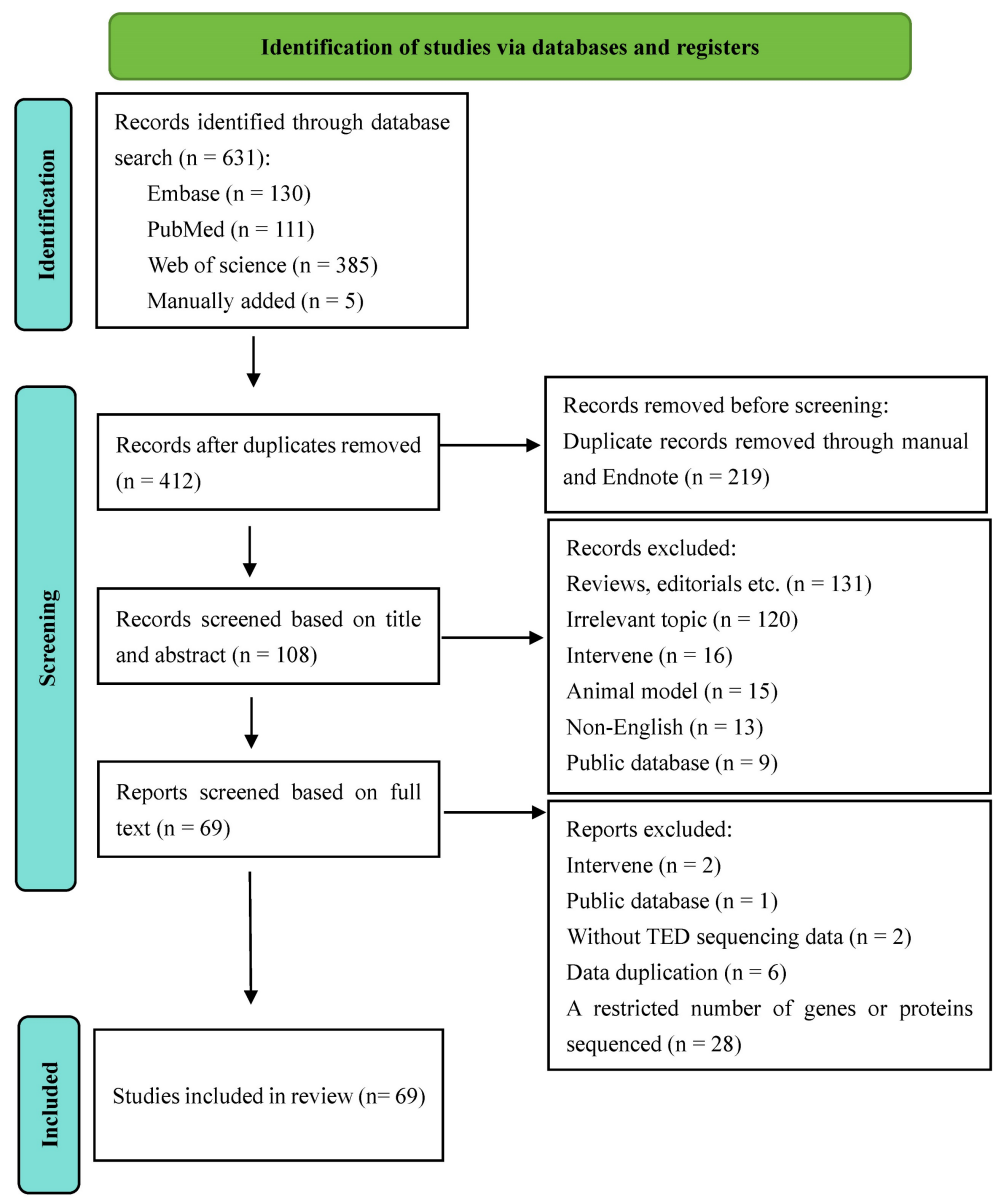
Flow Diagram of study selection - adapted from the flow diagram template provided by PRISMA 2020.

A total of 412 studies were screened using the described search strategy. At the end of the selection process, 69 studies were included in the systematic review. Details of the screening process are provided in the PRISMA flow diagram (Figure 1). Of the 69 studies, 4 studies (5.80%) utilized genomics and epigenomics, 33 studies (47.83%) utilized transcriptomics, 5 studies (7.25%) utilized metabolomics and lipidomics, 13 studies (18.84%) utilized proteomics, 7 studies (10.14%) utilized microbiomics, 6 studies (8.69%) utilized two omics assays, and 1 study (1.45%) utilized three omics assays. These studies were conducted between 2005 and 2024, with significant contributions from China, the UK, the US, Germany, Korea, and other countries. According to the quality assessment, 8 of the 69 studies received a score of "low" 54 received a score of "moderate" and 7 received a score of "high" Further information about this quality assessment is available in Supplementary Table 4.

A total of 69 studies involved 1,363 TED patients and 1,504 controls (including 1,007 healthy controls (HCs) and 497 patients with GD or other diseases). The patients included in this review exhibit low heterogeneity, with ages ranging from 30.0 to 70.3 years and an overall mean age of approximately 48.40 years. The distribution of sex was similar across most studies, with a total of 322 males and 776 females. Although most of the included studies have a higher number of female participants, 2 studies reported a significantly higher proportion of male patients (male/female ratio ≥ 1.5). This data aligns with population statistics for TED.

The included studies analyzed various subgroups of TED. Forty studies compared TED with HC. Nine studies compared TED, GD, and HC. Five studies compared TED and GD. Six studies compared active TED, inactive TED, and HC. Seven studies examined TED based on severity, smoking status, which is a risk factor for the development of TED, and other valuable factors. One study compared TED, nonspecific orbital inflammation, sarcoidosis, and granulomatosis with polyangiitis. Additionally, one study employed 16S rRNA gene sequencing and metabolic network-driven analysis to investigate the association between gut microbiota and TED-related traits.

### Genomics and Epigenomics

This systematic review included 5 studies employing genomics and epigenomics. Of these, 1 study (20.0%) utilized genomics, while 4 studies (80.0%) investigated DNA methylation. Among the studies, 3 (60.0%) used orbital tissues as samples, and 2 studies (40.0%) analyzed peripheral blood.

In the genomic and epigenomic analysis of TED, whole-genome sequencing (WGS) and DNA methylation assays were applied, yet no significant genetic variants were identified 30. For DNA methylation analysis, the sequencing approaches included whole-genome bisulfite sequencing (WGBS) 21, methylation microarrays 31, and reduced representation bisulfite sequencing (RRBS) 32, 33, DNA methylation data were analyzed using various approaches, including differential expression analysis to identify key genes 21, functional and pathway analysis with Gene Ontology (GO) and Kyoto Encyclopedia of Genes and Genomes (KEGG) for gene role classification 21, 31, network analysis to explore gene interactions 21, and epigenetic analysis techniques such as differentially methylated probes and differentially methylated region distribution to pinpoint methylation changes 31-33. Bioinformatics tools like Ingenuity were used for data integration, while statistical and machine learning methods, including MIRA score calculation and random forest analysis, were employed for pattern recognition and correlation analysis 33, DNA methylation analysis detected various biomarkers. Notably, genes related to inflammation, adipogenesis, and autoimmunity, such as PTPRU and VCAM-1, exhibited significant alterations in methylation levels, potentially playing a key role in the pathogenesis of TED 21, 31. Pathway analysis and MIRA scoring identified four biological pathways significantly associated with TED, with genes such as LDLR, CDK5, and PIK3CB correlating with the TED phenotype 33. Additionally, several genomic loci, including CD14, IL17RE, CDK5, DRD4, and ZCCHC6, exhibited significant differences in methylation patterns associated with TED incidence 32.

### Transcriptomics

This systematic review included 36 studies employing transcriptomics. Of these, 29 studies (77.8%) analyzed orbital tissues (orbital connective tissues in 27 studies, 75.0%, and extraocular muscles in 2 studies, 5.6%). Six studies (16.7%) used peripheral blood as the sample (peripheral blood mononuclear cells (PBMC) in 4 studies, 11.1%, and serum in 2 studies, 5.6%), and 1 study (2.8%) used thyroid tissue. Regarding sequencing methods, 16 studies (44.4%) utilized RNA-seq, 13 studies (36.1%) employed microarray, 4 studies (11.1%) used single-cell RNA sequencing (scRNA-seq), and 1 study (2.8%) implemented miRNA microarray. Additionally, nanostring and single-nucleus RNA sequencing (snRNA-seq) were each used in 1 study (2.8%). A wide range of data analysis methodologies were employed. DEGs were used in 31 studies (86.1%), KEGG pathway analysis in 20 studies (55.6%), GO analysis in 15 studies (41.7%), gene set enrichment analysis (GSEA) in 7 studies (19.4%), and protein-protein interaction (PPI) networks in 5 studies (13.9%). Cell-cell interaction analysis and principal component analysis (PCA) were each used in 2 studies (5.6%). Other methods, such as miRanda, competing endogenous RNA (ceRNA) network construction, and co-expression analysis, were employed in 1 study each (2.8%).

Most studies utilized a single approach—identifying DEGs in TED patients compared to HCs and conducting functional analysis using GO, KEGG, and GSEA. In studies analyzing orbital tissues, genes involved in antigen presentation, immune adhesion, interferon-γ (IFN-γ) signaling, and immune cell markers for macrophages, B cells, and T cells were found to be upregulated. Additionally, the relaxin signaling pathway, which regulates fibrosis in TED, was active in TED patients. Four studies reported dysregulation of Wnt signaling gene expression, including Wnt5a, secreted frizzled-related proteins, and Dickkopf-related proteins 19, 34-36. It is well established that the typical Wnt signal regulates the fate of neural crest progenitor cells. Similarly, activation of the Wnt/β-catenin pathway inhibits commitment to the adipocyte lineage while promoting differentiation into bone cells 37, 38. Two studies used scRNA-seq to analyze peripheral blood mononuclear cells (PBMCs) and found that CD169+ classical monocytes and a novel CD4+ cytotoxic T lymphocyte (CTL) subtype with chemotactic and inflammatory characteristics contributed to hyperinflammation, fibrosis, and adipogenesis in orbital tissues 39, 40. Additionally, Z Cheng *et al.* identified the VEGF-A gene as a regulator of the cytotoxic function of CD4+ CTLs in TED 41.

### Proteomics

This systematic review included 18 studies employing proteomics. Among these, 5 studies (27.8%) analyzed orbital tissues (orbital connective tissues in 4 studies, 80.0%, and extraocular muscles in 1 study, 20.0%). Four studies (22.2%) analyzed serum, and 9 studies (50.0%) used tear samples. Liquid chromatography-tandem mass spectrometry (LC-MS/MS) was the most used sequencing assay, employed in 11 studies (61.1%). Additionally, 2 studies (11.1%) utilized matrix-assisted laser desorption/ionization time-of-flight mass spectrometry (MALDI-TOF MS), while other studies (27.8%) used Nano LC/QTOF, surface-enhanced laser desorption/ionization time-of-flight mass spectrometry (SELDI-TOF-MS), and proximity extension assays.

Of the five studies that analyzed orbital tissues, four employed LC-MS/MS to examine differentially expressed proteins (DEPs) by comparing TED patients to HCs. These studies found that proteins involved in tissue inflammation, adipose tissue differentiation, and metabolism were typically overexpressed in TED patients 21, 23. These findings suggest a shift in glycometabolism and lipometabolism in TED orbital tissues, which may help elucidate the underlying pathological mechanisms of TED. In serum samples, DEPs analysis, along with KEGG, GO, and PPI analyses, revealed significantly increased levels of IL6, CSF1, FLT3LG, and C4A in TED patients. These inflammatory proteins may play a crucial role in the pathogenesis of TED and could serve as potential new biomarkers for clinical use 42, 43. Nine studies analyzed DEPs in tear samples, finding that protective proteins such as PROL1, PRP4, and β2-microglobulin were markedly downregulated, while inflammatory proteins such as lysozyme C and cystatin S were upregulated in the patient group compared to controls 44-47. Enrichment analysis of the DEPs indicated that pathways related to the immune system, apoptosis, cell cycle, carbohydrate metabolism, and protein synthesis and degradation may be key in TED patients.

### Metabolomics and Lipidomics

This systematic review included 9 studies employing metabolomics. Among these, 3 studies (33.3%) used targeted metabolomics, while 6 studies (66.7%) utilized non-targeted metabolomics, including 1 study (11.1%) that specifically used non-targeted lipidomic metabolomics. Of the 9 studies, 3 (33.3%) analyzed orbital connective tissues, 3 (33.3%) used serum, 1 study (11.1%) analyzed feces, 1 study (11.1%) employed tear samples, and 1 study (11.1%) examined both orbital connective tissues and serum.

The non-targeted lipidomic metabolomics study utilized nanoflow ultrahigh pressure liquid chromatography-electrospray ionization tandem mass spectrometry (nUPLC-ESI MS/MS) sequencing, with data analyzed using Student's t-test and PCA. This study identified significantly increased levels of sphingosine-1-phosphate in serum and urine samples, indicating its potential as a biomarker for TED diagnosis. Among the other 6 non-targeted metabolomics studies, 3 studies (50.0%) employed LC-MS sequencing, 2 studies (33.3%) used gas chromatography-time-of-flight mass spectrometry (GC-TOF MS), and 1 study (16.7%) utilized nuclear magnetic resonance (NMR) spectroscopy. Metabolite profiles in TED patients, including fumarate, proline, phenylalanine, and glycerol, suggested a potential metabolic connection between orbital connective tissues and blood metabolites. Notably, cholesterol metabolism was significantly linked to TED pathogenesis.

When comparing TED patients to HCs, metabolites such as short-chain fatty acids, uric acid, uracil, hexose-phosphate, and D-sedoheptulose 1,7-bisphosphate were enriched in TED samples. Combining TED-specific modulators—proline and 1,5-anhydroglucitol—with key metabolites—lycine, glycerol 3-phosphate, and estrone sulfate—substantially improved the biomarker model's ability to discriminate between HCs, GD, and TED groups. All 3 targeted metabolomics studies utilized LC-MS/MS sequencing, with data analyzed using factor analysis, linear regression models, and various univariate analyses. Metabolic profiling revealed significant upregulation of glycolysis-related parameters (F6P/F16BP, AMP/ATP, ADP/ATP, and lactate) in orbital fibroblasts of TED patients compared to HCs. Additionally, active TED patients showed an increased ratio of putrescine to ornithine and spermine in serum compared to inactive TED patients.

### Microbiomics

This systematic review included 8 studies that utilized microbiomics. Among these, 6 studies (75.0%) analyzed fecal microbiota, 1 study (12.5%) examined orbital adipose tissues, and 1 study (12.5%) investigated ocular microbiota. Of the studies, 6 (75.0%) employed 16S rRNA sequencing, while 2 studies (25.0%) used 16S rDNA sequencing. The methodologies for identifying biomarkers and analyzing co-occurrence patterns within the microbiota varied. Notably, α- and β-diversity indices were used in 5 studies (62.5%), and random forest analysis was employed in 3 studies (37.5%). Additionally, specific techniques such as KEGG, weighted gene co-expression network analysis (WGCNA), and module-trait association analysis were utilized in 1 study (12.5%).

The collective evidence from these studies suggests that gut microbiota may play a significant role in TED, with specific microbial imbalances potentially contributing to immune dysregulation and disease development. TED patients exhibit decreased bacterial community diversity but show increased proportions of *Actinobacteria*, *Bacillus*, *Brevundimonas*, and a higher *Firmicutes*-to-*Bacteroidetes* ratio compared to HCs 48, 49. Specific taxa, such as *Deinococcus-Thermus*, *Chloroflexi*, and various bacterial species, display differential abundance patterns between TED and GD, indicating disease-specific microbial signatures 50. Further characterization reveals distinct microbiome types unique to TED patients, with taxa like *Klebsiella* pneumoniae, *Paracoccus*, and *Hemophilus* correlating with disease severity 48, 51. Additionally, s_Prevotella_copri and f_Prevotellaceae show significant correlation with TRAb, suggesting their potential role in TED development 52. Furthermore, bacterial species such as *Bacteroides*, *Dialister*, and *Lactobacillus* exhibit associations with serum lipopolysaccharide-binding protein concentration, indicating their involvement in TED pathophysiology 53.

## Discussion

TED is an autoimmune inflammatory disorder of the orbit, characterized by inflammation and a range of symptoms that can impact vision and appearance. Despite the presence of clinical scoring systems, there remains a critical need for more objective biomarkers to improve early diagnosis, disease monitoring, and therapeutic efficacy evaluation. Recent advancements in high-throughput sequencing technology and the rapid accumulation of omics data have led to increased utilization of genomics, proteomics, and other omics approaches to identify novel targets and biomarkers. Our systematic review comprehensively analyzed studies that employed genomics, epigenomics, transcriptomics, proteomics, metabolomics, lipidomics, and microbiomics to identify potential biomarkers in various biological samples, including orbital tissues, peripheral blood, feces, and tears. The integration of multi-omics data has significantly advanced our understanding of the molecular mechanisms underlying TED and has facilitated the identification of new diagnostic and prognostic biomarkers.

Among the 69 articles included in our study, numerous biomarkers were identified across various mediums, including fecal microbiota, tear fluid, orbital tissues, blood, and thyroid tissues. Connective tissues and extraocular muscles are the primary sites of pathological processes in TED. Studies often focus on exploring disease-specific molecular changes such as inflammation, adipogenesis, and immune responses. For instance, through transcriptomic analysis, researchers have identified the upregulation of genes related to antigen presentation, immune cell adhesion, and interferon-γ signaling in TED patients 19, 36. Tear samples reflect the health status of the ocular surface. Proteomic analysis has revealed the downregulation of protective proteins and upregulation of inflammatory proteins, which may be associated with ocular surface inflammation in TED patients 54. Fecal samples are used to investigate the association between gut microbiota and TED, revealing specific microbial imbalances related to immune dysregulation and disease development 50.

Through scRNA-seq, blood samples are utilized to explore the connections between the peripheral immune landscape and TED, such as the activation of CD169^+^ classical monocytes, which may be pivotal in driving the autoimmune reactions characteristic of TED 39. Combining data from different biological samples can provide a more comprehensive understanding of disease mechanisms. For example, combining orbital tissue and blood samples can reveal both local and systemic changes. Samples that are easily obtainable (such as blood and tears) may aid in developing non-invasive diagnostic tools for disease monitoring and prognosis. Examining biological samples from various sources allows for a more thorough comprehension of TED, spanning from localized tissue effects to broader immune system impacts, ultimately guiding the identification of more effective biomarkers.

In omics analyses, the selection of appropriate control groups is crucial for elucidating biological differences. In this systematic review, many studies compared TED patients with HC and patients with GD to identify biomarkers associated with TED. Comparing TED patients to HC helps reveal disease-specific molecular alterations, with the HC group providing a baseline against which pathological changes in TED patients can be contrasted. For example, significant differences in gene expression, protein levels, metabolites, and microbiota composition between TED patients and HC may shed light on pathogenic mechanisms and disease progression. Conversely, comparisons with GD patients aid in distinguishing the specific manifestations of TED within the context of GD. Although GD is a commonly associated with TED, not all GD patients develop TED. Differentiating TED from GD helps identify molecular signatures unique to TED, which may be related to disease severity, inflammatory responses, or tissue remodeling. Additionally, some studies compared TED with other inflammatory conditions, such as non-specific orbital inflammation, sarcoidosis, and granulomatosis with polyangiitis, or grouped patients based on factors like smoking status and serum lipopolysaccharide-binding protein levels. These comparisons aim to uncover the unique pathological features and influencing factors of TED.

In exploring biomarkers related to TED, the choice of sequencing methods is pivotal, requiring a nuanced understanding of different biological molecular levels as described by the central dogma, which is a principle that describes the flow of genetic information within a biological system 16. The central dogma was initially proposed by Francis Crick in 1957 and has since been refined. It outlines the flow of genetic information from DNA to RNA to proteins, and the selection of sequencing technology should align with different stages of this information flow. This review includes various sequencing methods, such as WGS, RRBS, and RNA-seq. Each method has its technical advantages and limitations: WGS offers comprehensive genomic information but at a higher cost; RRBS is more cost-effective but provides a narrower scope of information; RNA-seq reveals changes in gene expression, which is crucial for understanding TED's molecular mechanisms. Mass spectrometry is a vital technique for high-throughput sequencing in proteomics. For instance, MALDI-TOF serves as a cost-effective, efficient, and precise analytical tool widely used for microbial species identification and protein profiling in both clinical and research settings. MALDI-TOF can rapidly and accurately identify bacteria or proteins by analyzing their unique molecular fingerprints, thus providing valuable insights into their biological functions and characteristics 55-57. Within the framework of the central dogma, combining transcriptomics and proteomics methods offers a comprehensive view from gene expression to changes at the protein and metabolite levels. This multi-omics approach elucidates the relationship between gene expression regulation and protein function, leading to a more thorough understanding of the molecular mechanisms underlying TED.

In the realm of TED research, the selection of an appropriate technological approach to address specific scientific inquiries is a critical step. Therefore, the choice of technology should be guided by specific research questions and available biological materials. For instance, while WGS provides comprehensive genomic information, not all studies require such exhaustive analysis 30; RRBS or targeted gene panels might suffice. Similarly, for researchers interested in immediate molecular responses, RNA-seq may be more appropriate than microarrays. Proteomics can elucidate alterations in protein expression associated with inflammation, adipose tissue differentiation, extracellular matrix remodeling, and metabolism in the orbital tissues of TED patients 21. Metabolomics can identify metabolic changes related to the pathogenesis of TED, such as alterations in cholesterol metabolism, which may be linked to the inflammatory and immune responses of the disease 24. Although microbiomics may not be directly involved in the traditional central dogma, it provides a new perspective for understanding the systemic aspects of TED.

Multi-omics sequencing has identified a range of potential biomarkers for TED, offering promising applications for disease identification, course prediction, and treatment response evaluation. For example, Ji *et al.* explored the diagnostic value of a multi-panel approach combining various biomarkers to differentiate between TED and GD patients 58. Their study demonstrated an area under the curve range of 0.845 to 0.935, suggesting that such multi-panel approaches could effectively identify individuals with TED. However, the clinical applicability of these findings depends on further validation in larger, more diverse cohorts to ensure their generalizability and reliability. Similarly, Zhang *et al.* used proteomic and miRNA analyses to identify 20 DEPs, including Zonulin, α-2 macroglobulin, β-2 glycoprotein 1, and fibronectin, which may play roles in diagnosis and prognosis 59. While clinical practice currently relies on markers such as TBII, TSHR-Ab, TSI and TSAb, additional potential biomarkers that could serve as adjuncts for TED confirmation and prognosis assessment remain to be identified and validated. Despite the progress made in biomarker identification through multi-omics sequencing, realizing their full clinical potential involves overcoming several challenges. Future research should focus on establishing well-defined cohorts, conducting longitudinal studies, and addressing technical, logistical, and economic barriers to the integration of these biomarkers into routine clinical practice.

Recent advancements in multi-omics technologies are enhancing the discovery and potential development of informative biomarkers for clinical practice 60. These technologies offer valuable insights into the complex pathophysiology of TED and facilitate the identification of new diagnostic and therapeutic biomarkers 17, 21, 61. Despite the promising prospects, several challenges remain, particularly regarding the translation of research discoveries into clinical applications. A significant issue is the inadequacy of study designs, which often results in low statistical significance and limits the impact of many research efforts. Future research in TED should continue to leverage multi-omics technologies, with a focus on improving sample collection, data standardization, and analytical methods 62. Additionally, it is crucial to validate the clinical relevance of these biomarkers through larger sample sizes and multicenter studies to ensure their robustness and applicability in clinical settings.

It is important to recognize the limitations and challenges associated with the current body of research. Firstly, there is a need to increase sample sizes to enhance the robustness of findings. Larger sample sizes will improve the statistical power and generalizability of results. Secondly, standardization of biomarker detection methodologies across studies is crucial. Such standardization ensures the reliability and reproducibility of results, facilitating more accurate comparisons and interpretations. Thirdly, the validation of identified biomarkers in independent cohorts remains inadequately addressed. Many studies, according to the QUADOMICS criteria, have used patient populations that are not fully representative, and there has been insufficient validatory estimation and assessment, which may lead to overfitting. Fourthly, the lack of quantitative analysis prevents the performance of a meta-analysis, as the available data is not sufficiently homogeneous to draw meaningful conclusions or make reliable statistical inferences. Future research should focus on overcoming these limitations by developing more representative patient cohorts, implementing standardized biomarker detection protocols, and rigorously validating biomarkers in independent studies. With continued research efforts, the integration of novel biomarkers holds the potential to significantly enhance disease management.

## Conclusion

This systematic review has explored the complex landscape of TED through the application of multi-omics sequencing, revealing a wide range of potential biomarkers that could significantly enhance diagnosis, monitoring, and treatment of this multifaceted condition. Despite variations in methodologies and the early stage of biomarker discovery, the evidence collectively highlights the transformative potential of genomics, transcriptomics, proteomics, metabolomics, lipidomics, and microbiomics in unraveling the intricate pathophysiology of TED. Our comprehensive analysis has demonstrated the value of diverse biological samples—such as orbital tissues, peripheral blood, feces, tears, and ocular microbiota—in providing insights into the molecular mechanisms of TED. These biomarkers reflect the systemic nature of the disease and underscore the potential for developing minimally invasive diagnostic approaches. The identification of upregulated genes and proteins associated with inflammation, adipogenesis, and immune dysregulation, along with unique metabolic and microbial signatures, presents promising avenues for future research of TED.

## Supplementary Material

Supplementary tables.

## Figures and Tables

**Table 1:** Detailed information of studies on genomics and epigenomics

Author	Year	Region	Sample types	Total sample size	TED			Control			Sequencing methods	Data analysis methods	Main findings
					Sample size	Gender (M/F)	Age (y)	Sample size	Gender (M/F)	Age (y)			
G Dottore *et al.*^30^	2021	Pisa, Italy	Orbital tissue (OF)	12	6	4/2	50.30 ± 12.50	6	4/2	65.20 ± 17.10	WGS	/	No gene variants were detected but the differentially expressed genes in TED are involved in the regulation of proliferation, apoptosis, and cell cycle, with an increase in global DNA methylation levels.
S Virakul *et al.*^21^	2021	Rotterdam, Netherlands	Orbital tissue (OF)	12	4 (Active)	1/3	63.50 ± 20.38	4	/	/	WGBS	Enrichment analysis of network, pathway and function	Active TED hypermethylated genes clearly linked to inflammation, while hypomethylated genes linked to adipogenesis and autoimmune-related genes.
					4 (Inactive)	/	/						
Y Liang *et al.*^31^	2021	Shanghai, China	Orbital tissue (Connective tissue)	8	4	1/3	35.25 ± 6.34	4	0/4	39.00 ± 7.85	WGBS	KEGG, GO, Co-analysis of genes in methylation and expression level	In those differentially methylated genes, PTPRU and VCAM-1 performed significant alteration in methylation level and potentially participated in pathogenesis.
Z Xin *et al.*^33^	2019	Beijing, China	Peripheral blood (PBMC)	12	6	0/6	54.5 (47-61)	6	0/6	56.2 (46-54)	RRBS	Enrichment analysis of pathway, methylation-based inference of regulatory activity score calculation, random forest analysis	Four significant pathways (Toxoplasmosis, Axon guidance, Focal adhesion, and Proteoglycans in cancer) were identified, with genes like LDLR, CDK5, and PIK3CB correlated with TED phenotype.
Z Xin *et al.*^32^	2017	Beijing, China	Peripheral blood (Plasma)	12	6	6/0	55.00 ± 3.87	6	6/0	51.00 ± 2.58	RRBS	GO, KEGG, PCA	Several genomic loci were identified to have significant differences in methylation patterns that were associated with TED incidence, such as CD14, IL17RE, CDK5, DRD4, and ZCCHC6.

OF: orbital fibroblast; EOM: extraocular muscle; PBMC: peripheral blood mononuclear cell; WGS: whole-genome sequencing; WGBS: whole-genome bisulfite sequencing; RNA-seq: RNA sequencing; RRBS: reduced representation bisulfite sequencing; KEGG: Kyoto encyclopedia of genes and genomes; GO: gene ontology; PCA: principal component analysis; TED: thyroid eye disease; GD: Graves' disease.

**Table 2:** Detailed information of studies on transcriptomics

Author	Year	Region	Sample types	Total sample size	TED			Control			Sequencing methods	Data analysis methods	Main findings
					Sample size	Gender (M/F)	Age (y)	Sample size	Gender (M/F)	Age (y)			
L Zhu *et al.*^63^	2020	Beijing, China	Orbital tissue (EOM)	17	7	2/5	/	10	/	/	RNA-seq	GO, KEGG	The total m6A content was significantly increased in TED patients and identified aberrant expression of m6A methylation regulators.
X Bai *et al.*^35^	2022	Changsha, China	Orbital tissue (Connective tissue)	12	6	0/6	36.00 ± 13.45	6	3/3	30.67 ± 9.22	RNA-seq	GO, KEGG	Wnt was dysregulated and C5 was elevated in inactive TED vs HC, speculating C5 may promote remodeling of the orbital tissue.
A Sun *et al.*^64^	2023	Guangzhou, China	Orbital tissue (Connective tissue)	18	12	4/8	41.50 ± 13.50	6	3/3	44.50 ± 12.80	RNA-seq	KEGG, PPI	Relaxin signaling pathway is an important regulatory pathway in TED fibrosis pathogenesis, probably regulated by the differential expression of COL1A1, MMP2, FOS, GNG4, and CREB5.
H Ye *et al.*^65^	2022	Guangzhou, China	Orbital tissue (Connective tissue)	12	6 (Type Ⅰ)	1/5	31.50 ± 7.60	/	/	/	RNA-seq	GO, KEGG, ceRNA network construction, PPI	Hsa_circ_0007006 may regulate major genes, such as MMP2 and COL1A1, through the relaxin signaling pathway to affect TED typing.
					6 (Type Ⅱ)	3/3	51.50 ± 10.10						
L Wu *et al.*^66^	2021	Shanghai, China	Orbital tissue (Connective tissue)	12	6	/	/	6	/	/	RNA-seq	GO, KEGG, multivariate analysis of transcript splicing	Differential expression transcript and differential alternative splicing genes were found in TED vs HC, associated with immune modulation, extracellular matrix remodeling and adipogenesis.
J Khong *et al.*^67^	2015	Victoria, Australia	Orbital tissue (Connective tissue)	54	12 (Active)	/	/	21	/	/	Microarray	GO, KEGG, GSEA	TIMD4, defensins (DEFA1, DEFA1B, and DEFA3) and markers for adipogenesis (SCD, FADS1, and SCDP1) were overexpressed in active TED vs inactive TED, associated with immune and inflammatory response.
					21(Inactive)	/	/						
L Wu *et al.*^68^	2020	Shanghai, China	Orbital tissue (Connective tissue)	6	3	/	/	3	/	/	RNA-seq	GO, KEGG, Co-expression and interaction analysis of circRNA-miRNA	The differentially expressed circRNAs might participate in pathogenesis of TED, and circRNA_14940-CCND1-Wnt signaling pathway might be an important regulatory axis.
Y Wang *et al.*^69^	2023	Wuxi, China	Orbital tissue (Connective tissue)	6	3	/	/	3	/	/	RNA-seq	PPI, GO, KEGG, GSEA	MAB21L1, PIK3C2G and CLVS2 were downregulated in TED vs HC, associated with dysregulation of differentiation, oxidative stress and developmental pathways.
L Wu *et al.*^70^	2021	Shanghai, China	Orbital tissue (Connective tissue)	6	3	/	/	3	/	/	RNA-seq	GO, KEGG, Co-expression analysis of lncRNA-mRNA	LncRNAs might regulate extracellular matrix remodeling in orbital tissue of TED. The target genes of lncRNAs involved in the lipid metabolism and cytokine-cytokine receptor interaction were identified. The negative correlations between lnc-PTP4A2-3:7-TNXB and LINC01139:4-SPON1 might be related to the altered extracellular matrix.
L Wu *et al.*^17^	2020	Shanghai, China	Orbital tissue (EOM)	10	5	/	/	5	/	/	Microarray	GO, KEGG	The expressions of genes involved in cell adhesion and ECM metabolism were significantly different between TED and HC.
M Chen *et al.*^18^	2008	Taipei, China	Orbital tissue (Connective tissue)	25	17	2/15	31.30 ± 6.20	8	2/6	41.00 ± 10.80	Microarray	GSEA	Lysosome-related genes, such as CLN2, CLN3 and HEXB, were downregulated in TED, may be involved in the pathogenesis of adipose tissue hypertrophy in TED.
P Mou *et al.*^71^	2018	Shanghai, China	Orbital tissue (Connective tissue)	15	10	/	/	5	/	/	RNA-seq	GO	PTX3 in orbital tissue and plasma was increased in TED vs HC, might playing a role in the pathogenesis of TED at least in the deposition of extracellular matrix.
W Tao *et al.*^34^	2017	Florida, US	Orbital tissue (Connective tissue)	9	5	/	38.22 ± 11.78	4	/	38.43 ± 9.16	RNA-seq	GO	In TED OASC, expression of early neural crest progenitor marker (Wnt signaling, ZIC genes and MSX2) was lost. Meanwhile, ectopic expression of HOXB2 and HOXB3 was found in the OASC from TED.
Z Li *et al.*^72^	2022	Guangzhou, China	Orbital tissue (Connective tissue)	20	10	/	/	10	/	/	scRNA-seq	Trajectory analysis, cell-cell interactions analysis, GO, KEGG	The study revealed the pro-inflammation and pro-adipogenesis role of LPFs with RASD1 expression. ACKR1^+^ ECs and ATMs with an M2 phenotype were also identified as playing a role in TED. CD8^+^CD57^+^ CTLs showed the terminal differentiation phenotype and high IFN-g gene expression.
B Lee *et al.*^73^	2018	California, US	Orbital tissue (Connective tissue)	6 (Active)	3	0/3	70.33 ± 7.93	3	0/3	66.00 ± 9.93	RNA-seq	GO, KEGG, enrichment analysis of drugs and diseases	The IL-5 and chemokine signaling pathways were highly enriched, and very-low-density-lipoprotein receptor activity and statin medications were implicated as having a potential role in TED.
D Ezra *et al.*^19^	2012	London, UK	Orbital tissue (Connective tissue)	9 (Active)	5	1/4	51.20 ± 12.37	4	/	/	Microarray	/	The highest-ranked differentially expressed genes were dominated by IGF-1 signaling genes and data also demonstrate dysregulation of Wnt signaling gene expression, including Wnt5a, sFRPs and DKK.
D Kim *et al.*^36^	2021	Maryland, US	Orbital tissue (Intraconal connective tissue)	6	3	1/2	46.33 ± 4.04	3	/	/	RNA-seq, single-nucleus RNA sequencing	KEGG, RNA velocity analysis, pseudotime analysis, regulon analysis, PCA	Signaling pathways including PI3K-Akt signaling, etc. are enriched in orbital fat isolated from patients with TED. The adipocyte-specific genes FABP4/5, APOE, PPARG and ADIPOQ during adipogenic differentiation differential expressed. The insulin-like growth factor-1 receptor and Wnt signaling pathways appear to be enriched early in adipogenesis.
T Planck *et al.*^20^	2014	Malmö, Sweden	Orbital tissue (Intraorbital connective tissue)	26	8 (TED smokers)	4/4	57.50 ± 9.35	5 (Healthy smokers)	0/5	/	Microarray	Enrichment analysis of pathway	Immediate early genes, IL-1B and IL-6 were overexpressed in smokers with severe active TED compared to nonsmokers, suggesting that smoking activates pathways associated with adipogenesis and inflammation.
					8 (TED nonsmokers)	1/7	60.50 ± 14.07	5 (Healthy nonsmokers)	0/5	/			
T Planck *et al.*^74^	2011	Malmö, Sweden	Orbital tissue (Intraorbital connective tissue)	30	10 (Active)	4/6	55.30 ± 11.25	10	1/9	52.90 ± 9.55	Microarray	Enrichment analysis of pathway	PTHLH, which can inhibit adipogenesis, is downregulated both in active and chronic TED, indicating the possibility of an increased risk of adipogenesis.
					10 (Chronic)	0/10	54.40 ± 12.55	10 (Chronic lymphedema)	1/9	52.90 ± 9.55			
M Lantz *et al.*^75^	2005	Mainz, Germany	Orbital tissue (Intraorbital connective tissue)	10	5	2/3	54.60 ± 10.16	5	/	/	Microarray	/	Adipocyte-related immediate early genes are overexpressed in active ophthalmopathy, and CYR61 may have a role in both orbital inflammation and adipogenesis and serve as a marker of disease activity.
J Rosenbaum *et al.*^76^	2015	Portland, US	Orbital tissue (Connective tissue)	86	25	/	51.62 (Mean)	20	/	61.00 (Mean)	Microarray	GSEA, PCA	Inflammatory markers are far less characteristic of TED relative to other orbital inflammatory diseases.
								25 (NSOI)	/	50.74 (Mean)			
								8 (GPA)	/	41.7 (Mean)			
								8 (Sarcoidosis)	/	49.09 (Mean)			
R Ma *et al.*^77^	2022	Shanghai, China	Orbital tissue (Connective tissue)	10	5	/	/	5	/	/	RNA-seq	KEGG	Multiomic analyses of glycolysis and mitochondrial metabolism (TCA & OXPHOS) suggested that a glycolytic shift may exist in TED.
S Jang *et al.*^78^	2016	Seoul, Korea	Orbital tissue (Connective tissue)	12	8	/	/	4	/	/	Microarray	/	The level of miR-146a expression was significantly higher in TED orbital adipose tissue than in non-TED.
S Kumar *et al.*^79^	2005	Minnesota, US	Orbital tissue (Connective tissue)	28	20	/	/	8	/	/	Microarray	/	Gene expression profiling identified sFRP-1 as another gene up-regulated in TED patient tissues.
J Kor *et al.*^80^	2023	Seoul, Korea	Orbital tissue (Connective tissue)	18	12	5/7	21-59	6	2/4	27-64	RNA-seq	GO, KEGG, GSEA	YAP expression in TED was enhanced. Response to mechanical stimulus-related genes were overexpressed in TED, along with those enriched for adipose proliferation, inflammatory responses and hormone stimulus responses.
S Fang *et al.*^8^	2019	Shanghai, China	Orbital tissue (Connective tissue)	6	3	/	/	3	/	/	scRNA-seq	/	Gene expression of the master transcription factors TBX21 and STAT1 (Th1 cell), GATA3 and STAT6 (Th2 cell), RORA and STAT3 (Th17 cell), and AHR (Th22 cell) revealed the possible involvement of various effector T cells in TED orbits.
Z Yue *et al.*^81^	2022	Shanghai, China	Orbital tissue (Connective tissue)	6	3	/	/	3	/	/	RNA-seq	GO, KEGG, target gene prediction	A total of 50 tRFs and 361 mRNAs were dysregulated in the TED group, and tRF5-GluCTC, PMAIP1, HSD17B2 and ATF3 were verified to be significantly differentially expressed in TED.
Z Yue *et al.*^82^	2021	Shanghai, China	Orbital tissue (Connective tissue)	6	3	/	/	3	/	/	Microarray	PPI, GO, KEGG, ceRNA network construction	Two important ceRNA pathways were identified, the LINC01820:13-hsa-miR-27b-3p-FPR2 ceRNA pathway and the ENST00000499452-hsa-miR-27a-3p-CXCL1 pathway, which probably affect the autoimmune response and inflammation in TED patients.
D Wang *et al.*^39^	2024	Guangzhou, China	Peripheral blood (PBMC)	9	5	/	/	4 (GD)	/	/	scRNA-seq	KEGG, pseudotime trajectory analysis, transcription factor analysis, cell-cell interactions analysis,	Monocytes along with CD169^+^ clas_mono-derived macrophages, contributed to hyperinflammation, fibrosis, and adipogenesis in orbital tissues. CD169^+^ clas_mono expansion is related to activated IFNg signaling pathway in TED.
N Kim *et al.*^83^	2023	Korea (Seongnam, Seoul)	Peripheral blood (Plasma)	15	5 (Active)	3/2	46.40 ± 11.20	5	3/2	39.60 ± 12.20	NanoString	KEGG	Ten circulating miRNAs were found to be differentially expressed, with miR-484 and miR-192-5p showing significantly lower expression levels in the active TED group compared to the inactive TED group.
					5 (Inactive)	3/2	43.00 ± 16.60						
L Zhang *et al.*^59^	2018	UK (Cardiff), Italy (Milan), Germany (Essen)	Peripheral blood (Plasma)	46	19	19/0	48.40 ± 10.60	13	13/0	47.2 ± 11.7	miRNA-seq	Lasso regression, KEGG, target gene prediction, PCA	The top known and novel miRNAs were hsa-mir-497, hsa-mir-320b-1 and hsa-mir-320b-2, up-regulated in GD, Novel:19_15038 and hsa-miR-27a-3p up-regulated in TED.
								14 (GD)	14/0	51.6 ± 17.9			
R Liu *et al.*^84^	2023	Shanghai, China	Peripheral blood (PBMC)	20	10	/	/	10	/	/	Microarray	Target gene prediction	MiR-376b, which is downregulated in TED patients' PBMCs and regulated by T3, might play a role in TED pathogenesis by influencing HAS2 and inflammatory factors.
Y Wang *et al.*^40^	2021	Xi'an, China	Peripheral blood (PBMC)	9	6	/	/	3 (GD)	/	/	scRNA-seq	GSEA, GO	The novel TED-specific cell type CD4^+^ cytotoxic T lymphocytes, which are characterized by chemotactic and inflammatory features, were characterized by cytotoxicity (GZMB, KLRG1, GZMH, PRF1, GNLY and FGFBP2), chemotaxis (CCL4, CCL5 and CX3CR1) and inflammation (IL-1B and IFNG).
Z Cheng *et al.*^41^	2023	Xi'an, China	Peripheral blood (PBMC)	40	20	/	/	20 (GD)	/	/	RNA-seq	PPI, GSEA	VEGC-A was identified as a regulator of the cytotoxic function of CD4^+^ cytotoxic T lymphocytes in TED.
L Wescombe *et al.*^85^	2009	Sydney, Australia	Thyroid	18	10	1/9	40.6 (20-57)	8 (GD)	0/8	28 (17-44)	Microarray	/	Two hundred and ninety-five genes were differentially expressed between patients with TED and GD. Of these, the cardiac calsequestrin gene was the most highly expressed gene in TED.
Y Huang *et al.*^9^	2022	Shanghai, China	Orbital tissue (Lacrimal Gland)	76	26 (Active)	13/13	48.92 ± 11.59	25	12/13	42.12 ± 11.66	RNA-seq	GO, KEGG	Increased expression of cytokines and chemokines accompanied by a variety of immune cell infiltrations mainly involving T cells, B cells, and monocytes was found in TED lacrimal glands. An in-depth investigation into T cell subsets revealed IFN-γ-producing Th1 and IL-17A-producing Th17 cell-dominated autoimmunity in the active TED lacrimal microenvironment.
					25 (Inactive)	9/16	41.84 ± 12.54						

EOM: extraocular muscle; PBMC: peripheral blood mononuclear cell; PPI: protein-protein interaction network; GSEA: ‌gene set enrichment analysis; scRNA-seq: single cell RNA sequencing; miRNA-seq: microRNA sequencing; NSOI: nonspecific orbital inflammation; GPA: granulomatosis with polyangiitis; TED: thyroid eye disease; GD: Graves' disease; GO: Gene Ontology.

**Table 3:** Detailed information of studies on proteomics

Author	Year	Region	Sample types	Total sample size	TED			Control			Sequencing methods	Data analysis methods	Main findings
					Sample size	Gender (M/F)	Age (y)	Sample size	Gender (M/F)	Age (y)			
S Virakul *et al.*^21^	2021	Rotterdam, Netherlands	Orbital tissue (OF)	14	4 (Active)	1/3	63.50 ± 20.38	5	0/5	61.20 ± 17.61	LC-MS/MS	Enrichment analysis of network, pathway and function	Proteins that are typically involved in inflammation, cellular proliferation, hyaluronan synthesis and adipogenesis, were overexpressed, while various proteins associated with extracellular matrix biology and fibrotic disease, were typically overexpressed.
					5 (Inactive)	0/5	48.20 ± 12.12						
L Wu *et al.*^17^	2020	Shanghai, China	Orbital tissue (EOM)	6	3	/	/	3	/	/	LC-MS/MS	GO, KEGG, GSEA, PPI	Proteins mainly related to the composition (such as MYH1) and contraction force (such as MYH3) of the muscle fibers were significantly up regulated in TED, as well as those (such as VCAN) associated with cell adhesion. Differentially expressed proteins related to the components and metabolism of ECM (such as COL1A1) were identified in EOM samples of TED.
K Cheng *et al.*^23^	2013	Kaohsiung, China	Orbital tissue (Connective tissue)	14	7	4/3	56.60 ± 9.70	7	2/5	57.60 ± 7.10	LC-MS/MS	/	Up-regulations of the specific proteins are associated with biochemical mechanisms or capacities against endoplasmic reticulum stress, mitochondria dysfunction, and cell proliferation as well as apoptosis in TED orbital fat tissues.
N Matheis *et al.*^86^	2015	Sweden (Malmö), Germany (Rhinel-Palatinate)	Orbital tissue (Connective tissue)	64	6 (Untreated nonsmokers)	1/5	30 (20 -73)	12 (Control orbital tissue)	4/8	67.2 (48-85)	MALDI TOF/TOF	/	Proteins involved in tissue inflammation, adipose tissue differentiation, lipid metabolism, and tissue remodeling were up regulated in orbital tissue of untreated patients with TED and steroids decreased the expression of these proteins, whereas smoking attenuated such effect.
					16 (Steroid-treated nonsmokers)	5/11	55.1 (20-76)	13 (Control peripheral tissue)	1/12	61 (16-78)			
					17 (Steroid-treated smokers)	7/10	48.5 (27-71)						
R Ma *et al.*^77^	2022	Shanghai, China	Orbital tissue (Connective tissue)	10	5	/	/	5	/	/	LC-MS/MS	KEGG	Multiomic analyses of glycolysis and mitochondrial metabolism (TCA & OXPHOS) suggested that a glycolytic shift may exist in TED.
J Kang *et al.*^42^	2021	Kunming, China	Peripheral blood (Plasma)	9	3	2/1	42-54	3	0/3	25-27	LC-MS/MS	GO, KEGG, PPI	MYH11, P4HB, and C4A were markedly upregulated in TED patients and have been reported to participate in apoptosis, autophagy, the inflammatory response, and the immune system.
								3 (GD)	1/2	34-50			
L Zhang *et al.*^59^	2018	UK (Cardiff), Italy (Milan), Germany (Essen)	Peripheral blood (Plasma)	46	19	19/0	48.40 ± 10.60	13	13/0	47.2 ± 11.7	Nano LC/QTOF	Lasso regression, KEGG	Proteomic and miRNA analyses identified circulating biomarkers, including Zonulin and Fibronectin, differentiating between GD, TED, and healthy controls, suggesting diagnostic and prognostic potential.
								14 (GD)	14/0	51.6 ± 17.9			
H Ueland *et al.*^43^	2022	Bergen, Norway	Peripheral blood (Plasma)	220	36	7/29	41 (19-70)	120	54/66	40 (23-68)	Proximity extension assay	Student's t-test or Mann-Whitney U-test, Spearman's correlation, ROC	Distinctly increased levels of IL6, CSF1, FLT3LG, and FGF21 were observed in TED, suggesting that these inflammatory proteins could be important in the pathogenesis, and therefore potential new biomarkers for clinical use.
								64 (GD)	16/48	43 (15-66)			
T Shi *et al.*^61^	2023	Beijing, China	Peripheral blood (Plasma)	60	30	16/14	46.50 ± 10.60	30	15/15	42.10 ± 9.00	LC-MS/MS	Lasso regression, KEGG, interaction analysis of metabolites and gene	CPS1, GP1BA, and COL6A1 were identified as feature proteins and had better prediction performance for TED compared to the baseline model.
T Shi *et al.*^87^	2022	Beijing, China	Tear (Exosomes)	64	24	9/15	45.20 ± 10.10	16	5/11	40.40 ± 9.60	LC-MS/MS	GO, KEGG	The levels of the exosomal proteins Caspase-3, complement C4A and APOA-IV were significantly increased in TED patients compared to GD patients and controls.
								24 (GD)	10/14	40.30 ± 8.90			
X Zhou *et al.*^88^	2022	Shanghai, China	Tear	60	30	8/22	42.00 ± 11.91	30	8/22	40.27 ± 9.32	LC-MS/MS	KEGG, GO	Up-regulated proteins were mainly enriched in lipid metabolism and platelet molecular function, and down-regulated proteins were involved in binding, cell junction, and cellular process.
C Aass *et al.*^47^	2016	Oslo, Norway	Tear	42	21	6/15	44 (26-69)	21 (GD)	4/17	57 (20-77)	LC-MS/MS	Spearman's correlation	Upregulation of lacrimal gland proteins such as lysozyme C, lacritin, antileukoproteinase and zinc-alpha-2-glycoprotein 1 suggests involvement of the lacrimal gland in the pathogenesis of TED.
N Matheis *et al.*^44^	2012	Mainz, Germany	Tear	60	45	8/37	50 (22-77)	15	2/13	45 (33-74)	SELDI-TOF-MS, MALDI-TOF-MS	Analysis of variance, ROC	PRP4 and β2-microglobulin was decreased while lysozyme C and cystatin S was upregulated in TED, demonstrating altered regulation of proinflammatory and protective proteins in tears of patients with TED.
L Jiang *et al.*^89^	2021	Shanghai, China	Tear	12	6	3/3	50.20 ± 5.40	6	2/4	46.70 ± 6.30	LC-MS/MS	PLS-DA, GO, KEGG, PPI, GRN	Signaling pathways of the immune system, apoptosis, cell cycle, cytoskeleton, metabolism of carbohydrates, cofactors and vitamins, protein synthesis and degradation, vesicle-mediated transport of membrane and proteins may play key roles in patients with inactive TED.
C Chng *et al.*^90^	2018	Singapore, Singapore	Tear	72	18 (Mild)	5/13	54.17 (Mean)	18 (AITD)	5/13	57 (Mean)	LC-MS/MS, SWATH-MS	Mann-Whitney U test, Kruskal-Wallis tests, geometric mean	Two proteins, S100A4 and PIP showed consistent dysregulation trends in the discovery and validation phase experiments, may serve as potential biomarkers to predict progression to severe TED in patients with AITD.
					18 (Severe)	11/7	56 (Mean)	18	8/10	58 (Mean)			
E Kishazi *et al.*^46^	2018	Lausanne, Switzerland	Tear	12	6	/	/	6	/	/	LC-MS/MS	/	Cystatin c (TED/controls ratio: 1.53), alpha-1 antichymotrypsin (ratio: 1.70) and retinal dehydrogenase (ratio: 0.68), displaying differential levels in the tears of TED patients as highly promising biomarkers after verification.
R. Okrojek *et al.*^91^	2009	Mainz, Germany	Tear	60	45	/	/	15	/	/	SELDI-TOF-MS	Multivariate discriminant analysis, ROC	A set of biomarkers in the mass range of 3000-20000 Da, showing significant differences. The corresponding ROC curve had an area under the curve of 0.99 and a specificity and sensitivity of over 90% each.
N Matheis *et al.*^45^	2015	Mainz, Germany	Tear	120	30	7/23	45.5 (17-68)	30	3/27	47.5 (21-70)	MALDI-TOF MS	Enrichment analysis of pathway	Upregulation of inflammatory proteins and downregulation of protective proteins (i.e., PROL1/PRP4).
					30 (TED + Dry eye)	5/25	51 (31-70)	30 (Dry eye)	7/23	54.5 (32-80)			

OF: orbital fibroblast; EOM: extraocular muscle; MALDI TOF/TOF: matrix-assisted laser desorption/ionization time-of-flight/ time-of-flight; Nano LC/QTOF: nanoscale liquid chromatography/quadrupole-time-of-flight; SELDI-TOF-MS: surface-enhanced laser desorption/ionization time-of-flight mass spectrometry; MALDI-TOF MS: matrix-assisted laser desorption/ionization time-of-flight mass spectrometer; SWATH-MS: sequential window acquisition of all theoretical spectra mass spectrometer; ROC: receiver operating characteristic; TED: thyroid eye disease; GD: Graves' disease; GO: Gene Ontology.

**Table 4:** Detailed information of studies on metabolomics and lipidomics

Author	Year	Region	Sample types	Total sample size	TED			Control			Sequencing methods	Data analysis methods	Main findings
					Sample size	Gender (M/F)	Age (y)	Sample size	Gender (M/F)	Age (y)			
F Biscarini *et al.*^27^	2023	UK (Wales, Newcastle, London), Italy (Milan, Pisa), Belgium (Brussels), Germany (Essen)	Fecal microbiota	146	10	/	/	11	/	/	NMR	PLS-DA	Short-chain fatty acids (SCFAs, butyrate, propionate etc.) was increased in TED vs HC.
								26 (GD)	/	/			
R Du *et al.*^22^	2023	Shanghai, China	Orbital tissue (Connective tissue)	12	7	2/5	33.80 ± 8.90	5	1/4	39.80 ± 8.90	LC-MS/MS	PLS-DA, enrichment analysis of pathway	In TED vs HC, the phosphoenolpyruvic acid was upregulated and downregulated metabolites were uric acid, phosphoenolpyruvic acid etc. and the TED-related pathways identified included purine metabolism, beta-alanine metabolism, glutathione metabolism.
J Huang *et al.*^24^	2022	Shanghai, China	Orbital tissue (Connective tissue)	23	11	3/8	54.63 ± 14.49	12	2/10	36.58 ± 13.05	LC-MS/MS	PLS-DA, KEGG, interaction analysis of metabolites and gene	TED has a significant difference in the metabolic profiles, altering cellular metabolome, especially cholesterol metabolism.
R Ma *et al.*^77^	2022	Shanghai, China	Orbital tissue (Connective tissue)	10	5	/	/	5	/	/	LC-MS/MS	/	Metabolic profiles provided evidence that the parameters favoring glycolysis (F6P/F16BP, AMP/ATP, ADP/ATP, and lactate) were upregulated in the TED.
D Ji *et al.*^58^	2018	Seoul, Korea	Orbital tissue (Connective tissue)	10	5	3/2	53.6 (Mean)	5	3/2	52.8 (Mean)	GC-TOF MS	/	The up-regulated metabolites included proline, fumarate, and phenylalanine that were consistent with the expression pattern in blood metabolite analysis. The activated tissue metabolism was represented by amino acids, including asparagine, valine, allo-threonine, methionine, and glycine.
D Ji *et al.*^58^	2018	Seoul, Korea	Peripheral blood (Plasma)	79	26	7/19	39.50 ± 10.30	32	6/26	/	GC-TOF MS	PLS-DA, ROC, enrichment analysis of pathway, PCA	With combination of proline and 1,5-anhydroglucitol, which were identified as TED-specific modulators, the re-constructed biomarker model greatly improved the statistical power and facilitated simultaneous discrimination among healthy control, TED, and GD without TED groups.
								21 (GD)	6/15	36.40 ± 10.40			
T Shi *et al.*^61^	2023	Beijing, China	Peripheral blood (Plasma)	60	30	16/14	46.50 ± 10.60	30	15/15	42.10 ± 9.00	LC-MS/MS	PLS-DA, lasso regression, KEGG, interaction analysis of metabolites and gene, PCA	Lycine, glycerol 3-phosphate, and estrone sulfate were identified as feature metabolites and had better prediction performance for TED compared to the baseline model.
S Byeon *et al.*^92^	2022	Seoul, Korea	Peripheral blood (Plasma)	86	31	8/23	/	32	6/26	/	nUPLC-ESI MS/MS	PCA	The sphingosine-1-phosphate levels showed consistent and significant increases in both plasma and urine samples of patients with TED.
								23(GD)	6/17	/			
H Ueland *et al.*^93^	2023	Bergen, Norway	Peripheral blood (Plasma)	200	36	8/23	/	100	23/77	39 (15-70)	LC-MS/MS	Linear regression models, factor analysis	No difference in systemic activation of the kynurenine pathway in GD patients with and without TED implies that the local Th1 immune response in the orbit is not reflected systemically.
								64 (GD)	6/17	/			
B Billiet *et al.*^54^	2022	Angers, France	Tear	45	21 (Active)	5/16	55 (Mean)	/	/	/	LC-MS/MS	/	Two short chain acylcarnitines, propionylcarnitine and butyrylcarnitine, and spermine showed increased concentrations and the ratio putrescine/ornithine, representing the activity of ornithine decarboxylase, was significantly increased in patients with active compared to inactive TED.
					24 (Inactive)	6/18	51 (Mean)						

NMR: nuclear magnetic resonance; LC-MS/MS: liquid chromatography-mass spectrometry/mass spectrometry; GC-TOF MS: gas chromatography-time of flight mass spectrometry; nUPLC-ESI MS/MS: nanoflow ultrahigh pressure liquid chromatography tandem mass spectrometry; PLS-DA: partial least squares-discriminant analysis; ROC: receiver operating characteristic; TED: thyroid eye disease; GD: Graves' disease.

**Table 5:** Detailed information of studies on microbiomics

Author	Year	Region	Sample types	Total sample size	TED			Control			Sequencing methods	Data analysis methods	Main findings
					Sample size	Gender (M/F)	Age (y)	Sample size	Gender (M/F)	Age (y)			
T Shi *et al.*^49^	2019	Beijing, China	Fecal microbiota	65	33	17/16	46.00 ± 11.71	32	16/16	43.40 ± 9.70	16S rRNA	α- and β-diversity indexes, random forest analysis, Mann-Whitney U test, LEfSe	Bacterial community diversity was significantly reduced in TED vs HC; *Bacteroidetes* was significantly increased in TED vs HC.
T Shi *et al.*^50^	2020	Beijing, China	Fecal microbiota	95	33	17/16	46.00 ± 11.70	32	16/16	43.40 ± 9.70	16S rRNA	α- and β-diversity indexes, random forest analysis, Mann-Whitney U test, KEGG	*Deinococcus-Thermus* and *Chloroflexi* were significantly reduced in TED vs GD; *Subdoligranulum* and *Bilophila* were increased in TED vs GD; Viral protein family was the only enrichment metabolic pathway in GD vs TED.
								30 (GD)	10/20	45.00 ± 12.80			
T Shi *et al.*^52^	2019	Beijing, China	Fecal microbiota	31	31	15/16	45.40 ± 11.80	/	/	/	16S rRNA	WGCNA, pearson correlation analysis, negative binomial regression, latent class analysis	The abundance of s_Prevotella_copri and f_Prevotellaceae was positively correlated with TRAb level.
Q Zhang *et al.*^51^	2023	Changsha, China	Fecal microbiota	80	20 (Mild)	7/13	37.50 ± 10.05	18	3/15	33.50 ± 10.60	16S rDNA	α- and β-diversity indexes, PCA, KEGG, target gene prediction	No remarkable difference in diversity in TED vs HC; The abundance of Klebisiella_Pneumoniae was positively correlated with disease severity.
					25 (Moderate-to-severe)	8/17	40.00 ± 14.45						
					17 (Sight-threatening)	12/5	55.00 ± 8.35						
F Biscarini *et al.*^27^	2023	UK (Wales, Newcastle, London), Italy (Milan, Pisa), Belgium (Brussels), Germany (Essen)	Fecal microbiota	146	46	6/40	47.04 ± 11.43	41	9/32	46.08 ± 13.45	16S rRNA	α- and β-diversity indexes, random forest analysis, pearson correlation analysis	*Actinobacteria* and F:B ratio were significantly increased and *Baceteroidetes* significantly decreased in GD/TED vs HC; *Bacteroides* abundance was positively correlated with TSH and negatively correlated with free thyroxine; Presence of *Clostridiales* at diagnosis was correlated with long-term TRAb persistence post antithyroid treatment.
								59 (GD)	6/53	46.39 ± 14.38			
A Fenneman *et al.*^53^	2023	Amsterdam, Netherlands	Fecal microbiota	15	8 (Low plasma levels of LBP)	2/6	42.12 ± 12.76	/	/	/	16S rRNA	Spearman correlation analysis	Plasma LBP level was positively correlated with Gram-negative gut bacteria abundance (*Baceteroides spp.*, *Dialister spp.*); *Lactobacillus spp.* was strongly increased in H-LBP vs L-LBP.
					7 (High plasma levels of LBP)	3/4	51.43 ± 9.31						
Y Li *et al.*^94^	2022	Beijing, China	Orbital tissue (Connective tissue)	54	27	9/18	48 (22-74)	27	17/10	25 (3-67)	16S rRNA	α-diversity indexes, PCA, co-occurrence network, multiple linear regression and mixed effect model	Four microbiome types were identified in orbital fat, one of which was exclusive to TED patients. A higher proportion of *Pseudomonas* in TED, but a lower proportion of *Enhydrobacter*. *Flavobacterium* was positively correlated with chemosis.
X Ji *et al.*^48^	2022	Taiyuan, China	Ocular microbiota	69	47	8/39	44.25 ± 13.44	22	7/15	62.64 ± 7.11	16S rDNA	α- and β-diversity indexes, spearman correlation analysis	The average relative abundance of *Bacillus* and *Brevundimonas* increased significantly in the TED group. *Corynebacterium* had a significantly decreased relative abundance. *Paracoccus*, *Hemophilus*, *Lactobacillus*, and *Bifidobacterium* were positively correlated with the severity of clinical manifestations or disease activity.

LBP: lipopolysaccharide-binding protein; 16S rRNA: 16S ribosomal RNA; 16S rDNA: 16S ribosomal DNA; LEfSe: linear discriminant analysis effect size; WGCNA: weighted gene co-expression network analysis; GD: Graves' disease.
