# Supplementary material for: Multi-Omics Approaches to Discover Biomarkers of Thyroid Eye Disease: A Systematic Review
Source: Int J Biol Sci. 2024 Nov 11;20(15):6038–55. doi: 10.7150/ijbs.103977 (PMC11628329; doi:10.7150/ijbs.103977)
Supplement: Supplementary file 1 — Supplementary tables. [file ijbsv20p6038s1.pdf]

Supplementary Materials

Multi-Omics Approaches to Discover Biomarkers of Thyroid Eye Disease: A Systematic Review

SUPPLEMENTARY MATERIALS-- 1

Supplementary Table 1. The search strategy for PubMed.---2

Supplementary Table 2. The search strategy for Embase.-- 3

Supplementary Table 3. The search strategy for Web of science.---4

Supplementary Table 4. Quality assessment of included studies using the QUADOMICS Tool.--- 5

Supplementary Table 1. The search strategy for PubMed.

Search	PubMed Query	Results
#4	#1 AND (#2 OR #3)	111
#3	"Sequence Analysis" OR "High-Throughput Nucleotide Sequencing" OR "RNA-Seq" OR "Oligonucleotide Array Sequence Analysis" OR "Sequence Analysis, DNA" OR "Sequence Analysis, Protein" OR "Gene Expression Profiling" OR "Single-Cell Gene Expression Analysis" OR "Mass Spectrometry" OR “Gas Chromatography-Mass Spectrometry OR “Liquid Chromatography-Mass Spectrometry” OR “Spectrometry, Mass, Electrospray Ionization” OR “Spectrometry, Mass, Matrix-Assisted Laser Desorption-Ionization” OR “Tandem Mass Spectrometry”	955,410
#2	"Multiomics"[Mesh] OR "Genomics"[Mesh] OR "Epigenomics"[Mesh] OR "Methylation"[Mesh] OR "Proteomics"[Mesh] OR "Microbial Consortia"[Mesh] OR "Gastrointestinal Microbiome"[Mesh] OR "Microbiota"[Mesh] OR "Metabolomics"[Mesh] OR "Transcriptome"[Mesh]	415,711
#1	"Graves Ophthalmopathy"[MeSH Terms] OR "Graves Ophthalmopathy"[Title/Abstract] OR "Thyroid Associated Ophthalmopathies"[Title/Abstract] OR "Thyroid Eye Disease"[Title/Abstract] OR "Thyroid Eye Diseases"[Title/Abstract] OR "Thyroid-Associated Ophthalmopathy"[Title/Abstract] OR "Thyroid Associated Ophthalmopathy"[Title/Abstract] OR "Dysthyroid Ophthalmopathy"[Title/Abstract] OR "Dysthyroid Ophthalmopathies"[Title/Abstract] OR "Graves Eye Disease"[Title/Abstract] OR "Graves Orbitopathy"[Title/Abstract] OR "Myopathic Ophthalmopathy"[Title/Abstract] OR "Congestive Ophthalmopathy"[Title/Abstract] OR "Edematous Ophthalmopathy"[Title/Abstract]OR"Infiltrative Ophthalmopathy"[Title/Abstract]	5,928

Supplementary Table 2. The search strategy for Embase.

Search	Embase Query	Results
#4	#1 AND (#2 OR #3)	130
#3	'sequence analysis':ti,ab,kw OR 'bisulfite sequencing':ti,ab,kw OR 'DNA sequencing':ti,ab,kw OR 'high throughput sequencing':ti,ab,kw OR 'RNA sequencing':ti,ab,kw OR 'single cell RNA seq':ti,ab,kw OR 'whole exome sequencing':ti,ab,kw OR 'mass spectrometry':ti,ab,kw OR 'laser desorption ionization mass spectrometry':ti,ab,kw OR 'liquid chromatography-mass spectrometry':ti,ab,kw OR 'tandem mass spectrometry':ti,ab,kw OR 'time of flight mass spectrometry':ti,ab,kw OR 'time resolved mass spectrometry':ti,ab,kw	658,213
#2	'multiomics'/exp OR 'genome'/exp OR 'epigenome'/exp OR 'methylome'/exp OR 'microbial genome'/exp OR 'proteomics'/exp OR 'metabolomics'/exp OR 'transcriptome'/exp	642,438
#1	'endocrine ophthalmopathy'/exp OR 'dysthyroid eye disease':ti,ab,kw OR 'dysthyroid ophthalmopathy':ti,ab,kw OR 'dysthyroid orbitopathy':ti,ab,kw OR 'endocrine orbitopathy':ti,ab,kw OR 'graves eye disease':ti,ab,kw OR 'graves ophthalmopathy':ti,ab,kw OR 'graves orbitopathy':ti,ab,kw OR 'graves` eye disease':ti,ab,kw OR 'graves` ophthalmopathy':ti,ab,kw OR 'graves` orbitopathy':ti,ab,kw OR 'ophthalmic graves disease':ti,ab,kw OR 'ophthalmic graves` disease':ti,ab,kw OR 'ophthalmopathy, endocrine':ti,ab,kw OR 'thyroid associated eye disease':ti,ab,kw OR 'thyroid associated ophthalmopathy':ti,ab,kw OR 'thyroid associated orbitopathy':ti,ab,kw OR 'thyroid eye disease':ti,ab,kw OR 'thyroid ophthalmopathy':ti,ab,kw OR 'thyroid orbitopathy':ti,ab,kw OR 'thyroid related orbitopathy':ti,ab,kw OR 'endocrine ophthalmopathy':ti,ab,kw	9,597

Supplementary Table 3. The search strategy for Web of science.

Search	Web of Science Query	results
#4	#1 AND (#2 OR #3)	385
#3	TS= (Sequence Analysis OR High-Throughput Nucleotide Sequencing OR RNA-Seq OR Oligonucleotide Array Sequence Analysis OR Gene Expression Profiling OR Single-Cell Gene Expression Analysis OR Mass Spectrometry OR Gas Chromatography-Mass Spectrometry OR Liquid Chromatography-Mass Spectrometry OR Electrospray Ionization Mass Spectrometry OR Matrix-Assisted Laser Desorption-Ionization Mass Spectrometry OR Tandem Mass Spectrometry)	3,671,791
#2	TS= (Multiomics OR Genomics OR Epigenomics OR Methylation OR Proteomics OR Microbial Consortia OR Gastrointestinal Microbiome OR Microbiota OR Metabolomics OR Transcriptome)	1,413,269
#1	TS= (Graves Ophthalmopathy) OR TI=(Graves Ophthalmopathy OR Thyroid Associated Ophthalmopathies OR Thyroid Eye Disease OR Thyroid Eye Diseases OR Thyroid-Associated Ophthalmopathy OR Thyroid Associated Ophthalmopathy OR Dysthyroid Ophthalmopathy OR Dysthyroid Ophthalmopathies OR Graves Eye Disease OR Graves Orbitopathy OR Myopathic Ophthalmopathy OR Congestive Ophthalmopathy OR Edematous Ophthalmopathy OR Infiltrative Ophthalmopathy) OR AB=(Graves Ophthalmopathy OR Thyroid Associated Ophthalmopathies OR Thyroid Eye Disease OR Thyroid Eye Diseases OR Thyroid-Associated Ophthalmopathy OR Thyroid Associated Ophthalmopathy OR Dysthyroid Ophthalmopathy OR Dysthyroid Ophthalmopathies OR Graves Eye Disease OR Graves Orbitopathy OR Myopathic Ophthalmopathy OR Congestive Ophthalmopathy OR Edematous Ophthalmopathy OR Infiltrative Ophthalmopathy)	13,471

Supplementary Table 4. Quality assessment of included studies using the QUADOMICS Tool.

Author	Year	1	2	3	4.1	4.2	5	6	7	8	9	10	11	12	13	14	15	16	Score	Quality
G Dottore et al.	2021	Yes	No	Yes	Yes	Yes	Yes	Yes	Yes	Yes	Yes	No	Yes	No	Yes	No	Yes	No	11	Mod
S Virakul et al.	2021	Yes	No	Yes	No	No	Yes	Yes	Yes	Yes	Yes	Yes	Yes	No	Yes	No	Yes	No	11	Mod
L Zhu et al.	2020	Yes	No	Yes	No	No	Yes	Yes	Yes	Yes	Yes	Yes	No	No	Yes	No	Yes	No	10	Mod
Y Liang et al.	2021	Yes	No	Yes	Yes	Yes	Yes	Yes	Yes	Yes	Yes	Yes	Yes	No	Yes	No	Yes	No	12	Mod
Z Xin et al.	2019	Yes	No	Yes	Yes	No	Yes	Yes	Yes	Yes	Yes	Yes	Yes	No	Yes	No	Yes	No	12	Mod
Z Xin et al.	2017	Yes	No	Yes	Yes	No	Yes	Yes	Yes	Yes	Yes	Yes	Yes	No	Yes	No	Yes	No	12	Mod
A Fenneman et al.	2023	Yes	Yes	Yes	Yes	Yes	Yes	Yes	Yes	Yes	Yes	Yes	Yes	No	Yes	Yes	Yes	Yes	15	High
X Bai et al.	2022	Yes	No	Yes	No	No	Yes	Yes	Yes	Yes	Yes	Yes	Yes	No	Yes	No	Yes	No	11	Mod
A Sun et al.	2023	Yes	No	Yes	No	No	No	Yes	Yes	Yes	Yes	No	Yes	No	Yes	No	Yes	No	9	Mod
H Ye et al.	2022	Yes	No	Yes	No	No	No	Yes	Yes	Yes	Yes	Yes	Yes	No	Yes	No	Yes	No	10	Mod
L Wu et al.	2021	Yes	No	Yes	No	Yes	No	Yes	Yes	Yes	Yes	Yes	Yes	No	Yes	No	Yes	No	11	Mod

J Khong et al.	2015	Yes	No	Yes	No	No	Yes	No	Yes	Yes	Yes	Yes	Yes	No	Yes	No	Yes	No	10	Mod
L Wu et al.	2020	Yes	No	Yes	No	Yes	Yes	Yes	Yes	Yes	Yes	Yes	Yes	No	Yes	No	Yes	No	12	Mod
Y Wang et al.	2023	Yes	Yes	Yes	Yes	No	Yes	Yes	Yes	Yes	Yes	Yes	Yes	No	Yes	Yes	Yes	No	14	High
L Wu et al.	2021	Yes	No	Yes	Yes	Yes	No	Yes	Yes	Yes	Yes	Yes	Yes	No	Yes	No	Yes	No	11	Mod
L Wu et al.	2020	Yes	No	Yes	No	Yes	Yes	Yes	Yes	Yes	Yes	Yes	Yes	No	Yes	No	Yes	No	12	Mod
M Chen et al.	2008	Yes	No	Yes	Yes	No	Yes	Yes	Yes	Yes	Yes	No	Yes	No	Yes	No	Yes	No	11	Mod
P Mou et al.	2018	Yes	Yes	Yes	Yes	Yes	Yes	Yes	Yes	Yes	Yes	Yes	No	No	Yes	Yes	Yes	No	13	High
W Tao et al.	2017	Yes	No	Yes	No	No	Yes	Yes	Yes	Yes	Yes	Yes	No	No	Yes	No	Yes	No	10	Mod
Z Li et al.	2022	Yes	No	Yes	No	No	Yes	Yes	Yes	Yes	Yes	Yes	No	No	Yes	No	Yes	No	10	Mod
B Lee et al.	2018	Yes	No	Yes	Yes	Yes	Yes	Yes	Yes	Yes	Yes	Yes	Yes	No	Yes	No	Yes	No	12	Mod
D Ezra et al.	2012	Yes	No	Yes	No	No	Yes	No	Yes	Yes	Yes	Yes	No	No	Yes	No	Yes	No	9	Mod
D Kim et al.	2021	Yes	No	Yes	No	No	Yes	Yes	Yes	Yes	Yes	Yes	No	No	Yes	No	Yes	No	10	Mod
T Planck et al.	2014	Yes	No	Yes	Yes	No	Yes	Yes	Yes	Yes	Yes	Yes	No	No	Yes	No	Yes	No	11	Mod

T Planck et al.	2011	Yes	No	Yes	Yes	No	Yes	Yes	Unclear	Unclear	Unclear	Yes	No	No	Yes	No	Yes	No	8	Mod
M Lantz et al.	2005	Yes	No	Yes	Unclear	No	Yes	Yes	Unclear	Unclear	Unclear	Yes	No	No	Yes	No	Yes	No	7	Low
J Rosenbaum et al.	2015	Yes	No	Yes	Yes	Yes	Yes	Yes	Unclear	Unclear	Unclear	No	No	No	Yes	No	Yes	No	7	Low
R Ma et al.	2022	Yes	No	Yes	Yes	Yes	Yes	Yes	Yes	Yes	Yes	Yes	Yes	No	Yes	No	Yes	No	12	Mod
S Jang et al.	2016	Yes	No	Yes	Yes	No	Yes	Yes	Unclear	Unclear	Unclear	Yes	No	No	Yes	No	Yes	No	8	Mod
S Kumar et al.	2005	Yes	No	Yes	No	No	Yes	No	Unclear	Unclear	Unclear	Yes	No	No	Yes	No	Yes	No	6	Low
J Kor et al.	2023	Yes	No	Yes	No	No	Yes	Yes	Unclear	Unclear	Unclear	Yes	No	No	Yes	No	Yes	No	7	Low
S Fang et al.	2019	Yes	No	Yes	Yes	Yes	Yes	Yes	Unclear	Unclear	Unclear	Yes	No	No	Yes	No	Yes	No	8	Mod
Z Yue et al.	2022	Yes	No	Yes	Yes	Yes	Yes	Yes	Unclear	Unclear	Unclear	Yes	No	No	Yes	No	Yes	No	8	Mod
Z Yue et al.	2021	Yes	No	Yes	Yes	Yes	Yes	Yes	Unclear	Unclear	Unclear	Yes	No	No	Yes	No	Yes	No	8	Mod
D Wang et al.	2024	Yes	No	Yes	No	No	Yes	Yes	Yes	Yes	Yes	Yes	Yes	No	Yes	No	Yes	No	11	Mod
N Kim et al.	2023	Yes	No	Yes	Yes	No	Yes	Yes	Yes	Yes	Yes	Yes	Yes	No	Yes	No	Yes	No	12	Mod
L Zhang et	2018	Yes	Yes	Yes	Yes	No	Yes	Yes	Yes	Yes	Yes	Yes	Yes	No	Yes	Yes	Yes	No	14	High

al.																				
R Liu et al.	2023	Yes	No	Yes	Yes	No	Yes	Yes	Yes	Yes	Yes	Yes	Yes	No	Yes	No	Yes	No	12	Mod
Y Wang et al.	2021	Yes	No	Yes	Yes	Yes	Yes	Yes	Yes	Yes	Yes	Yes	Yes	No	Yes	No	Yes	No	11	Mod
Z Cheng et al.	2023	Yes	No	Yes	No	No	Yes	Yes	Unclear	Unclear	Unclear	Yes	No	No	Yes	No	Yes	No	11	Mod
L Wescombe et al.	2009	Yes	No	Yes	No	No	Yes	Yes	Yes	Yes	Yes	Yes	Yes	No	Yes	No	Yes	No	10	Mod
Y Huang et al.	2022	Yes	Yes	Yes	Yes	Yes	Yes	Yes	Yes	Yes	Yes	Yes	Yes	No	Yes	Yes	Yes	No	12	Mod
F Biscarini et al.	2023	Yes	Yes	Yes	Yes	Yes	Yes	Yes	Yes	Yes	Yes	Yes	Yes	No	Yes	Yes	Yes	No	11	Mod
R Du et al.	2023	Yes	No	Yes	No	Yes	Yes	Yes	Yes	Yes	Yes	No	Yes	No	Yes	No	Yes	No	10	Mod
J Huang et al.	2022	Yes	No	Yes	Yes	Yes	Yes	Yes	Yes	Yes	Yes	Yes	Yes	No	Yes	No	Yes	No	12	Mod
D Ji et al.	2018	Yes	No	Yes	No	No	Yes	Yes	Unclear	Unclear	Unclear	Yes	No	No	Yes	No	Yes	No	14	High
T Shi et al.	2023	Yes	Yes	Yes	Yes	Yes	Yes	Yes	Yes	Yes	Yes	Yes	Yes	No	Yes	Yes	Yes	No	11	Mod
S Byeon et al.	2022	Yes	Yes	Yes	Yes	Yes	Yes	Yes	Yes	Yes	Yes	Yes	Yes	No	Yes	Yes	Yes	No	12	Mod
H Ueland	2023	Yes	Yes	Yes	Yes	Yes	Yes	Yes	Yes	Yes	Yes	Yes	Yes	No	Yes	Yes	Yes	No	11	Mod

et al.																				
B Billiet et al.	2022	Yes	Yes	Yes	Yes	Yes	Yes	Yes	Yes	Yes	Yes	Yes	Yes	No	Yes	Yes	Yes	No	13	High
K Cheng et al.	2013	Yes	No	Yes	No	No	Yes	Yes	Yes	Yes	Yes	No	Yes	No	Yes	No	Yes	No	10	Mod
N Matheis et al.	2015	Yes	No	Yes	No	Yes	Yes	Yes	Yes	Yes	Yes	Yes	Yes	No	Yes	No	Yes	No	10	Mod
J Kang et al.	2021	Yes	No	Yes	No	No	Yes	Yes	Yes	Yes	Yes	Yes	Yes	No	Yes	No	Yes	No	12	Mod
H Ueland et al.	2022	Yes	Yes	Yes	Yes	No	Yes	No	Yes	Yes	Yes	Yes	Yes	No	Yes	Yes	Yes	No	9	Mod
X Zhou et al.	2022	Yes	No	Yes	Yes	Yes	Yes	Yes	Yes	Yes	Yes	Yes	Yes	No	Yes	No	Yes	No	10	Mod
C Aass et al.	2016	Yes	No	Yes	No	Yes	Yes	Yes	Yes	Yes	Yes	Yes	Yes	No	Yes	No	Yes	No	11	Mod
N Matheis et al.	2012	Yes	No	Yes	Yes	Yes	Yes	Yes	Yes	Yes	Yes	Yes	Yes	No	Yes	No	Yes	Yes	8	Mod
L Jiang et al.	2021	Yes	Yes	Yes	Yes	Yes	Yes	Yes	Yes	Yes	Yes	Yes	Yes	No	Yes	Yes	Yes	No	7	Low
C Chng et al.	2018	Yes	No	Yes	Yes	Yes	Yes	Yes	Yes	Yes	Yes	Yes	Yes	No	Yes	No	Yes	Yes	7	Low
E Kishazi et al.	2018	Yes	Yes	Yes	Yes	Yes	Yes	Yes	Yes	Yes	Yes	Yes	Yes	No	Yes	Yes	Yes	No	12	Mod
T Shi et al.	2022	Yes	Yes	Yes	Yes	Yes	Yes	Yes	Yes	Yes	Yes	Yes	Yes	No	Yes	Yes	Yes	No	8	Mod

R Okrojek et al.	2009	Yes	No	Yes	Yes	No	Yes	Yes	Unclear	Unclear	Unclear	Yes	No	No	Yes	No	Yes	No	6	Low
N Matheis et al.	2015	Yes	No	Yes	Yes	Yes	Yes	Yes	Yes	Yes	Yes	Yes	Yes	No	Yes	No	Yes	No	7	Low
T Shi et al.	2019	Yes	Yes	Yes	Yes	Yes	Yes	Yes	Yes	Yes	Yes	Yes	Yes	No	Yes	Yes	Yes	Yes	8	Mod
T Shi et al.	2020	Yes	Yes	Yes	Yes	Yes	Yes	Yes	Yes	Yes	Yes	Yes	Yes	No	Yes	Yes	Yes	Yes	8	Mod
T Shi et al.	2019	Yes	Yes	Yes	Yes	Yes	Yes	Yes	Yes	Yes	Yes	Yes	Yes	No	Yes	Yes	Yes	No	8	Mod
Q Zhang et al.	2023	Yes	Yes	Yes	Yes	Yes	Yes	Yes	Yes	Yes	Yes	Yes	Yes	No	Yes	Yes	Yes	No	11	Mod
Y Li et al.	2022	Yes	Yes	Yes	No	Yes	Yes	Yes	Yes	Yes	Yes	Yes	Yes	No	Yes	Yes	Yes	No	12	Mod
X Ji et al.	2022	Yes	Yes	Yes	Yes	Yes	Yes	Yes	Yes	Yes	Yes	Yes	Yes	No	Yes	Yes	Yes	No	14	High

Index: Item 1. Were selection criteria clearly described? 2. Was the spectrum of patients representative of patients who will receive the test in practice? 3. Was the type of sample fully described? 4. Were the procedures and timing of biological sample collection concerning clinical factors described in enough detail? 4a. Clinical and physiological factors 4b. Diagnostic and treatment procedures 5. Were handling and pre-analytical procedures reported in sufficient detail and similar for the whole sample? And, if differences in procedures were reported, was their effect on the results assessed? 6. Is the time period between the reference standard and the index test short enough to reasonably guarantee that the target condition did not change between the two tests? 7. Is the reference standard likely to correctly classify the target condition? 8. Did the whole sample or a random selection of the sample receive verification using a reference standard of diagnosis? 9. Did patients receive the same reference standard regardless of the result of the index test? 10. Was the execution of the index test described in sufficient detail to permit replication of the test? 11. Was the execution of the reference standard described in sufficient detail to permit its replication? 12. Were the index test results interpreted without knowledge of the results of the reference standard? 13. Were the reference standard results interpreted without knowledge of the results of the index test? 14. Were the same clinical data available when test results were interpreted as would be available when the test is used in practice? 15. Were uninterpretable/intermediate test results reported? 16. Is it likely that the presence of over-fitting was avoided; Yes=criteria achieved, represented in green; N=criteria not achieved, represented in red; Unclear= ambiguous or not stated, represented in yellow. studies that achieved 13/16

or more on the QUADOMICS tool were classified as ‘High’, whereas those that scored 9/16 or lower were classified as ‘Low’, and the rest were classified as ‘Mod’.
